# Mutations That Matter: Domain-Specific Mutational Hotspots in *BRCA1* and Its Key Interactors Differ Between TCGA and BCGA Breast Cancer Cohort—An In Silico Analysis

**DOI:** 10.3390/cancers18193083

**Published:** 2026-09-23

**Authors:** Mithila Kulkarni, Katheeja Muhseena Neeraje, Ranajit Das, Suparna Laha

**Affiliations:** 1 Cell Biology and Molecular Genetics Division (CBMG), Yenepoya Research Centre (YRC), Yenepoya (Deemed to be University), Deralakatte, Mangalore 575018, India; mithilakulkarni68@gmail.com (M.K.); katheejamuhseenan1994@gmail.com (K.M.N.); 2 Department of Psychiatry and Behavioral Science, UT Health, Houston, TX 77054, USA; 3 Data Analytics, Bioinformatics and Structural Biology (DABS), Yenepoya Research Centre (YRC), Yenepoya (Deemed to be University), Deralakatte, Mangalore 575018, India; ranajitdas@yenepoya.edu.in

**Keywords:** pathogenic variants, PALB2, RAD50, RB1, BARD1, domain-specific mutation clustering, population-specific genomics

## Abstract

Background: Patients with breast cancer (BC), and mostly triple-negative BC (TNBC), often exhibit poor prognosis due to variability in symptom manifestation and differential responses to treatment. TNBC among Indian women is the highest globally, contributing to increased mortality. A deeper understanding of the molecular factors influencing BC severity is essential for improving the therapeutic strategies. Objective: This study investigates differences in mutation patterns and positions in BRCA1 and its interacting partners, which contribute to variability in cancer manifestation and pathogenic burden. Methods: Mutation analysis is performed using publicly available cancer genomics datasets. Independent cross-cohort comparisons were performed using the non-overlapping datasets to ensure multi-layered independent validation of the analysis. Additionally, multi-database validation was performed to confirm the consistency of pathogenic and likely pathogenic variants. The FDR-adjusted *p*-value was used to assess the statistical significance of the difference in pathogenicity between the two cohorts. To minimize domain-length bias and avoid false enrichment signals, domain-length normalization was implemented alongside the chi-square test, enabling accurate assessment of mutation enrichment. Results: Functional domains differ in their impact on the disease manifestation. Pathogenic mutations cluster in specific functional domains of BRCA1 and its interactors, with distinct patterns between BCGA and TCGA cohorts. These differences support the view that both mutation type and positional context shape pathogenic outcomes and highlight demographic variability in disease profiles. Conclusion: This study emphasizes that domain-specific mutation clustering is associated with increased pathogenic burden. Hence, consideration of protein-domain context may provide additional information beyond gene-level mutation to optimize stratification and treatment outcomes. The inclusion of the BCGA dataset provides novel population-specific insights, representing one of the first domain-level mutational analyses in an Indian breast cancer cohort.

## Simple Summary

Breast cancer remains one of the leading causes of cancer-related mortality worldwide, with particularly aggressive forms, such as triple-negative breast cancer, disproportionately affecting younger women in India. A key driver of breast cancer risk is a mutation in the *BRCA1* gene and in several proteins that work alongside it, including PALB2, RAD50, BARD1, and RB1. These proteins form a molecular network that normally detects and repairs DNA damage; when mutated, this network of interacting complexes breaks down, and cells can become cancerous. In this study, we investigated the significance of the position of mutations, whether the specific location of a mutation within the functional domains of these proteins influences pathogenic outcomes, and finally, whether such domain-level mutational patterns vary demographically. Using publicly available genomic databases and computational analyses, we found that pathogenic mutations tend to concentrate within specific functional domains of each protein, and that the identity and location of these mutations differ substantially between the TCGA cohort (a large, US-based multi-ancestry cancer genomics dataset) and the BCGA cohort (an Indian breast cancer genomics resource). Notably, mutations at identical positions in the same gene were sometimes associated with different levels of disease pathogenicity, depending on the type of amino acid change and the cohort. These findings highlight that the precise position of a mutation within a protein’s functional architecture, rather than gene-level mutation status alone, can be a critical determinant of pathogenic outcome. The cohort-specific mutational landscapes may warrant distinct diagnostic considerations. This exploratory, in silico analysis provides a foundation for future functional and clinical validation studies aimed at improving precision medicine for breast cancer.

## 1. Introduction

Cancer is a highly heterogeneous disease, characterized by the absence of firmly established molecular events that drive its subtypes and prognosis. Recent investigations have identified specific proteins that serve as modifiers of *BRCA1*-associated cancers and play a role in modulating cancer risk [1]. *BRCA1* is involved in multiple protein complexes that play crucial roles in maintaining genomic integrity and preventing tumorigenesis [2]. It typically resides alongside BRCA2, BARD1, and RAD51, as well as key DNA damage repair proteins, BRIP1, forming a nuclear multi-protein complex [3,4] that detects abnormal DNA structures, such as double-strand breaks (DSBs) and base pair mismatches, and coordinates the DNA repair mechanism [5,6].

Mutations in *BRCA1* likely impair the cell’s ability to repair damaged DNA, rendering it susceptible to cancerous transformation [7,8,9]. Approximately 17% of index patients with breast cancer (BC) have pathogenic *BRCA1/2* germline mutations [10,11]. Loss of function of *BRCA1/2* due to a single allelic mutation leads to a lifetime risk of 40% and 18% for ovarian cancer (OC), respectively [12]. Genetic mutations in the *BRCA1/2* genes are possibly essential in explaining the inheritance pattern of some cancers, such as BC and OC, in certain families [2,3,13]; however, they may not fully account for interpersonal variations in age-specific cancer risk. Emerging research suggests that the modification of specific loci affects the heritability of BC in *BRCA1* mutation carriers [1]. These loci encode the domains of the proteins that interact with BRCA1, influencing DNA damage and repair pathways. Notably, genes encoding proteins that directly interact with BRCA1/2 have emerged as potent modifiers [1]. These proteins may modulate cancer risk in *BRCA1* mutation carriers [1]. In hereditary BC and OC, approximately 20% of cases are associated with pathogenic variations in *BRCA1/2*, while 5–10% are linked to other susceptibilities involving genes such as *TP53*, *BARD1*, *PALB2*, *RAD51*, *STK11*, *PTEN*, *ATM*, and *CHEK2* [2,10,14]. Recent investigations suggest that *BRCA1* mutations located within the RING and BRCT domains, as well as in the 11–13 exon regions, are necessary for the functioning of *BRCA1* and serve as binding sites for BRCA1-interacting proteins, such as RAD50, RAD51, BRCA2, PALB2, c-Myc, and pRb [15,16]. In addition, there are cases where severity develops in the absence of any BRCA mutation, such as in 1.2% with *PALB2* germline mutations, 1.5% with *ATM*, 2.5% with *CHEK2*, and 0.3% with the remaining proteins such as CH1, NBN, RAD51C, RAD51D, and TP53 [10,17,18]. Mutations in *PALB2* are directly linked to the risk of BC, as they develop a flaw in the BRCA1-PALB2-BRCA2 complex. Studies have found that a lower level of PALB2 mRNA expression is correlated with poor survival in patients with cancer [19]. Mutations in another interactor, BRIP1, also increase the risk of BC and OC [20,21]. The RB1 protein is crucial for controlling cell cycle progression, maintaining genomic stability, regulating apoptosis, and facilitating cellular differentiation [22]. When the *RB1* gene undergoes mutation or deletion, these regulatory processes are compromised, resulting in abnormal cell growth, proliferation, and tumor development [23,24,25,26]. Gene alterations of *RB1* are present in up to 40% of triple-negative breast cancers [27]. Based on these findings, we hypothesized that BRCA1-interacting partners influence cancer severity. Mutations in *BRCA* and its partners that disturb their interactions and compromise genetic integrity increase disease severity. The primary focus of this study was to establish the role of genes beyond *BRCA1* in the development and progression of polygenic diseases, such as cancer, by analyzing the change in the mutation pattern with demography, which finally led to alterations in the disease prognosis. Among various cancers, breast cancer is mostly dependent on *BRCA1*. It is the most prevalent malignancy among women worldwide. In India, approximately 100,000 women are diagnosed with breast cancer annually, with a case fatality rate of 40% [28,29,30], indicating that India has become a nation with a high number of breast cancer-related deaths [30,31]. Among the subtypes of BC, triple-negative breast cancer (TNBC) is mostly dependent on genetic mutations, particularly common in younger women, and represents the most aggressive form of breast cancer, with poor prognosis and high recurrence rates [32]. Moreover, in Western countries, TNBC accounts for 12.2–13% of all breast cancer cases, with the highest prevalence observed in African American populations (22.5–23.7%) [33]. Findings reveal that the prevalence of TNBC among Indian women is the highest (27.9–31%) compared to the prevalence among those in other regions worldwide and exhibits a distinct pattern with more aggressive features [34]. The higher incidence of TNBC, along with its complexity, may lead to a greater proportion of aggressive cases that are clinically challenging to treat, contributing to increased mortality [35]. The highest incidence of TNBC (27.9%) in India, compared to that in other regions worldwide, and its aggressive nature necessitate the need to study the mutational profile of the BCGA breast cancer patients compared to the TCGA breast cancer population. TNBC is characterized by the accumulation of both germline susceptibility variants and somatic genomic alterations, with BRCA1 pathway defects representing one important molecular mechanism. Hence, the primary focus of this study is on a specific set of molecules beyond *BRCA1* that are known *BRCA1* tumor suppressor gene modifiers, and to establish their role in the development and progression of the disease by analyzing the change in the mutation pattern with demography, which finally led to alterations in the disease prognosis. Recent large-scale genomic studies in Indian cohorts have revealed unique population-specific mutation patterns, including a higher frequency of BRCA1 185delAG, highlighting the need for region-specific diagnostic panels rather than reliance on Western TCGA-based benchmarks [36]. Our research involves the mutational analysis of *BRCA1* and its interacting molecules, specifically *PALB2*, *RAD50*, *BARD1*, and *RB1*, which are associated with pathogenicity and genomic instability linked to breast cancer. In the current research, we identified regions with observed mutation clustering within the domains of *BRCA1*-interacting proteins that correlated with pathogenic variant enrichment and were most likely to be associated with disease severity. Furthermore, we have compared the mutation profiles of the *BRCA1*-interacting genes and the significant changes in the position of the mutations in the BCGA and TCGA populations. This information can aid in gaining insights into cancer pathogenesis and provide potential targets for future population-based therapeutic interventions. We note here that this study does not compare somatic and germline mutation biology. Rather, it integrates somatic mutation distributions from cancer genomics cohorts with independent clinical pathogenicity annotations to investigate domain-specific mutation clustering in BRCA1 and its interacting proteins.

## 2. Materials and Methods

### 2.1. Exon–Domain Mapping of BRCA1 and Its Interacting Genes in Breast Cancer

This study focused on the mutation analysis of *BRCA1* and its interacting genes *PALB2*, *RAD50*, *BARD1*, *and RB1* in breast cancer patients, followed by a gene-specific comparative analysis of the mutations in different populations. To lay the groundwork for mapping exons and domains of these proteins (Table 1), which is essential for subsequent analyses, we extracted their gene and protein sequences, exon counts, and domain information from the NCBI database https://www.ncbi.nlm.nih.gov/, accessed on 14 September 2026 (Appendix A Table A1). mRNA sequences from NCBI were used to align amino acid sequences for detailed exon and domain mapping, integrating manual domain-to-exon mapping through literature and database reviews, which highlights the position of the mutation and predicts its effects (Appendix A Table A1). Mutation and gene expression data were sourced from the COSMIC database (https://cancer.sanger.ac.uk/cosmic, accessed on 14 September 2026), focusing on somatic mutations and expression levels across 13 cancer types with notable mutation frequencies and over-expression. This approach clarified the roles of these genes in cancer pathogenesis.

### 2.2. Multi-Layered Independent Validation Through Integrated Multi-Cohort Genomic Analysis

An in-depth cancer genomics analysis with breast cancer samples was conducted using cBioPortal (https://www.cbioportal.org/, accessed on 14 September 2026), which facilitated the visualization and examination of multidimensional data from TCGA patient samples and the demographically different BCGA cohort. The TCGA dataset includes mutation details of the samples, protein alterations, and clinical variability. We selected TCGA Pan-Cancer and TCGA-BRCA as our primary discovery cohorts over available institutional cohorts, because they provide exome-wide, multi-platform profiling of largely untreated primary tumors with harmonized survival endpoints, enabling unbiased exon- and domain-level hotspot detection. In contrast, the MSK panel-sequenced cohorts are optimized for clinically selected, often advanced cases and treatment-response questions, making them less suitable as a genome-wide reference for hotspot discovery in comparison with the population-level BCGA dataset. This comprehensive approach enabled the calculation of the mutation burden in breast cancer samples, which leads to a deeper understanding of the genomic landscape. Additionally, for independent cross-cohort validation to enhance the robustness and population specificity of the findings, a comparative mutation analysis between the pan-cancer TCGA dataset, demographically restricted to the USA (Caucasian, a smaller group of African American, Asian), and the breast cancer–specific BCGA (https://bcga.iitm.ac.in/cbioportal/, accessed on 14 September 2026) cohort comprising Indian cancer genomics data from IITM 2025, along with Chennai Breast Cancer Centre, Cancer Research and Relief Trust, and Karakinos Healthcare, was conducted. We note here that TCGA was not used as the proxy for homogeneous Western ancestry and, since BCGA consists of a small number of Indian samples, is not representative of large and highly heterogeneous Indian populations. We therefore summarize that this comparison was a cohort-level comparative analysis (TCGA vs. BCGA), and not a direct ancestry-matched population study (Western vs. South Asian). These datasets differ in population structure, sample origin, and data generation pipelines, thereby serving as independent validation cohorts. The reproducibility of domain-specific mutation enrichment across these cohorts supports the robustness of our findings. This study utilized curated somatic mutation data and clinical metadata from cBioPortal and related repositories to understand tissue-specific mutational patterns, thus providing a comprehensive, interconnected framework linking gene selection, mutation profiling, genomic analysis, and population-focused mutation mapping. Notably, no somatic vs. germline mutation comparison was performed, and no conclusion was drawn regarding this. This analysis will shed light on understanding the different grades of pathogenicity in BC between the TCGA and BCGA cohorts, which mostly come from different populations. The reproducibility of domain-specific mutation enrichment across these cohorts supports the robustness of our findings. The mutation enrichment in a particular gene was assessed by the occurrence of mutations at the FDs of that particular gene and scored using the following formula: occurrence of mutation = (number of samples having mutations in functional domain)/(total sample size)].

### 2.3. Multi-Database Validation of Pathogenicity

Validation of pathogenicity was performed using a multi-database approach integrating ClinVar and COSMIC annotations. ClinVar served as the primary source for clinical interpretation, with variants classified as pathogenic or likely pathogenic considered clinically pathogenic, while variants annotated as variant of uncertain significance (VUS), likely benign, or benign were analyzed separately and were not included in the pathogenic category. COSMIC and cBioPortal were used as an independent catalogue of cancer mutations to identify recurrent tumor-associated variants and mutation frequencies, but were not used as the sole source for clinical pathogenicity assignment. Where annotations differed between databases, ClinVar classifications were prioritized, whereas COSMIC was used to support evidence of somatic occurrence and recurrence in breast cancer. For pathogenicity analyses, individual variants were classified according to their available clinical annotations, with ClinVar serving as the primary source for variant-level interpretation. Variants classified as pathogenic (P) or likely pathogenic (LP) were grouped as clinically annotated pathogenic variants, irrespective of the cohort in which they were observed. Importantly, ClinVar classifications were assigned to individual variants and were not considered cohort-specific; therefore, the same exact variant was assigned the same pathogenicity category regardless of whether it was observed in the TCGA or BCGA cohort. Variants lacking a pathogenic or likely pathogenic ClinVar classification were not considered pathogenic for this analysis, but were not automatically classified as non-pathogenic or benign, as the absence of a P/LP annotation does not establish benignity. Variants explicitly classified as benign or likely benign were considered separately. Thus, differences between TCGA and BCGA were evaluated in terms of the observed distribution and frequency of specific variants and clinically annotated pathogenic variants, rather than differences in the pathogenicity classification of the same variant between cohorts. Pathogenic and likely pathogenic variants identified through this integrated annotation strategy were subsequently mapped to their corresponding exons and functional domains to identify the regions with observed mutation clustering and to compare the domain-specific mutation patterns between the TCGA and BCGA cohorts. This integrated methodological framework combines multi-database variant annotation, exon–domain mapping, and comparative cohort analysis to provide a comprehensive characterization of the mutational landscape of *BRCA1* and its interacting genes in breast cancer, while minimizing annotation bias through independent cross-validation of variant classifications.

The genomic analyses presented in this study primarily examined the distribution, recurrence, and domain-specific localization of mutations identified in publicly available cancer genomics resources. Somatic mutation datasets from TCGA, BCGA, and COSMIC were used to characterize mutation frequencies, positional clustering, and functional domain enrichment across breast cancer cohorts. Independently, ClinVar annotations were incorporated solely to provide the clinical interpretation of individual variants (e.g., pathogenic or likely pathogenic) and were not used to infer somatic mutation frequency, recurrence, or hotspot enrichment. Consequently, somatic mutation profiling and germline pathogenicity assessment were treated as distinct yet complementary analytical components throughout the study. No direct comparison between somatic and germline mutations was performed, and no conclusions regarding differences between these mutation classes were drawn.

### 2.4. Normalization-Based Validation by Domain-Length Normalized Mutation Burden (Mutations per kb) Analysis

To account for differences in functional domain size and minimize length-dependent inflation of mutation frequencies, domain-length normalization was performed for all functional domains (FDs) and non-characterized regions of BRCA1, PALB2, RAD50, BARD1, and RB1. Mutation density was calculated by dividing the average number of mutations within each domain by its corresponding coding length (kilobases), yielding normalized mutation density values expressed as the total number of mutations per kilobase (mutations/kb). Because these values represent mutation density rather than percentages, they may exceed a hundred mutations per kilobase and should not be interpreted as percentages. Domain-length normalization was performed independently for the TCGA breast cancer subset and the BCGA cohort to facilitate direct comparison of intrinsic mutational density across domains while accounting for differences in domain length.

Formula for mutation density calculation of a gene: (percentage of mutations in a specific domain of that gene)/(domain coding length in kb).

Domain-length normalization was used solely for descriptive comparison of mutation density and visualization of mutation enrichment and was not used directly for statistical hypothesis testing.

### 2.5. Statistical Analysis

Statistical comparisons between the TCGA and BCGA cohorts were performed using a Chi-square test applied to the observed mutation counts within the functional domain. FDR-adjusted *p*-value was used to assess the statistical significance of pathogenicity between the two cohorts. The odds ratio and relative risk were calculated independently for each functional domain. We note here that gene-specific domain comparisons were done independently between the two cohorts, and the cohorts were not compared across multiple genes or domains at the same time.

## 3. Results

### 3.1. Significant Accumulation of Mutations in BRCA1 and Its Interacting Genes in Different Cancer Types

One of the major causes of tumorigenesis is mutation. The accumulation of these mutations into clonal mutations leads to changes in gene expression patterns and various metabolic pathways, resulting in genomic instability [37,38]. We analyzed the mutation status and expression of the genes that have a role in maintaining genomic integrity in various cancer types from the patient sample data extracted from the COSMIC database. The bar graphs in (Figure 1A–F) illustrate the percentages of mutations in the oncogene *BRCA1* and its interactors across various cancer samples (CSs). We observed a marked increase in mutations in *BRCA1* and its interacting genes in the CSs, which are directly involved in the DNA damage sensing and repair processes. These essential functions may be altered due to the accumulation of mutations as observed in the CSs. On the contrary, the mutations in the housekeeping genes were found to be low compared to *BRCA1* and its interacting proteins. The housekeeping proteins are involved in basic cellular functions, such as cell cycle regulation, DNA replication, and cellular metabolism [39], which remain relatively unchanged due to the fewer mutations observed. These observations suggest that a major alteration of genetic integrity happens during cancer due to mutations in *BRCA1* and its interactors. In contrast, the basic cellular processes maintained by the housekeeping genes remain unaltered. Upon gene expression analysis, we observed a decrease in the expression of the *BRCA1* interactors in different CSs (Figure 1G–K). The mutation and expression were analyzed for *GAPDH* as a control housekeeping gene compared to the oncogene (Figure 1F).

### 3.2. The Mutations in Cancer Conditions Are Mostly in the Functional Domains of the Key Genes

To further understand the impact of the position of mutations in cancer development, we compared the occurrence of mutations in the functional domains (FDs) and the unknown domains (UKDs) of the selected genes in CSs screened from the cBioPortal database, considering two independent datasets, the TCGA and BCGA, with sample sizes of 10,967 and 973, respectively. The analysis of the TCGA samples (Supplementary data Sheet 1) showed that the occurrence of mutations in FDs in cancer types is higher compared to the UKDs. In the case of all cancer types, the FDs of the *BRCA1*, *PALB2*, *RAD50*, *BARD1* and *RB1* genes had high mutation burdens of 83.2%, 100%, 99.6%, 98.6% and 65.5%, respectively, compared to the UKDs, with mutation burdens of only 16.8%, 1.4% and 34.4% in *BRCA1, BARD1 and RB1* genes, respectively (Figure 2A). As BRCA and its interactors play a notable role in breast cancer, we were interested in analyzing their mutational distributions in breast cancer conditions. In the case of BC in the TCGA cohort, with a sample size of 4937 (Supplementary data Sheet 1), the profile changes marginally compared with all cancers. No mutations in the *BARD1* gene in the case of BC in the TCGA cohort were observed. The mutations in other genes like *BRCA1*, *PALB2*, *RAD50*, and *RB1* were mostly in the FDs with 74.1%, 100%, 100%, and 55.5%, respectively, which is similar to the profile in all cancers. The mutations in the UKDs were few; only *BRCA1* and *RB1* had mutations, at 18.5% and 44.4%, respectively, in UKDs (Figure 2B). Interestingly, in the case of the BCGA cohort, with a BC sample size of 494 (Supplementary data Sheet 1), the mutation rates are 100% in the FDs of *PALB2*, *RAD50*, and *BARD1*, and *BRCA1* has around 98% mutations in the FD. In the *RB1* gene, the occurrence of mutations in the UKD has come down to 24.5% compared to 44.4% in the TCGA cohort. The FD of *RB1* contains 72.5% mutations (Figure 2C). From this analysis, we understand that the FD has a comparatively higher percentage of mutations irrespective of cancer types, and the mutation burden differs between different genes. We also observed that in breast cancer, in the *BARD1* gene, there are no mutations in the (TCGA) population; in contrast, there are mutations in the (BCGA) population. So, in multiple genes, the occurrence of mutations was markedly higher in FDs compared to adjacent non-characterized regions, reinforcing domain-specific vulnerability.

### 3.3. Distribution of Mutational Burden Across the Domains of BRCA1 and Its Interacting Genes in TCGA and BCGA Cohorts Differs Demographically

An important question that emerges is whether the position of a mutation within different functional domains influences breast cancer severity. Furthermore, do differences in the mutational profiles of these genes contribute to variations in disease severity between the BCGA and TCGA cohorts? To establish this, we analyzed the distribution of mutations across the domains in the breast cancer population of the TCGA and BCGA cohorts. We studied the distribution of mutations/kb in the FD of breast cancer patients in the TCGA and BCGA cohorts using TCGA and BCGA datasets, which revealed that the mutation density profiles of FDs markedly differed between the two datasets (Supplementary data Sheet 2). Statistical significance was determined using a Chi-square test to compare the density of mutations within the domains of specific genes between the two datasets (Figure 3). In the *BRCA1* gene, both TCGA and BCGA cohorts exhibited a high mutation burden in the RD with 47.6 and 39.4 mutation density, respectively. In contrast, the BRCT-AD and BRCT2D displayed a profound difference between the cohorts. In the case of BRCT-AD, the TCGA cohort showed a higher mutation density of 12.1, while the BCGA cohort recorded a mutation density of 4.4. Inversely, in BRCT2D, the mutation density was higher in the BCGA cohort compared to TCGA, with 18.9 and 4.2, respectively (Figure 3A). The difference in the mutation profile across the domain of BRCA1 between the two cohorts was statistically significant (*p* = 0.003). Analysis of *PALB2* revealed an extremely significant population-specific difference in mutation density in the functional domain (*p* < 0.0001). The TCGA cohort exhibited mutation densities of 54.9 and 140 in C-CD and KEAP1D, respectively, whereas it was zero and 19.7, respectively, in the BCGA cohort. Conversely, the BCGA cohort showed a mutation density of 24.1 in the MRG15BD, which is markedly absent in the TCGA cohort (Figure 3B). In the DNA damage-sensing *RAD50* gene, the TCGA cohort demonstrated higher mutation density in the ATPaseD (12.1) and C-CD (26.8) regions compared to the BCGA. The BCGA cohort showed a higher burden of mutations at ZincD only, with a mutation density of 29.3 (Figure 3C). The cohort-specific mutation density difference was statistically significant in Rad50 as well, with a *p*-value of 0.0001. A marked difference was observed in the mutation status of the *BARD1* gene. While no breast cancer mutation data were available for the TCGA cohort within the studied sample size (*n* = 4937), the BCGA population exhibited an exceptionally high mutation burden concentrated in the RD with 70.7 mutation density (Figure 3D). The distinct difference in the BARD1 mutation profile between the two cohorts cannot be explained statistically due to the absence of any mutation of BARD1 in TCGA cohort. The *RB1* gene mutation profile showed that TCGA samples were highly enriched in D-C (111.4 mutation density), a finding markedly lower in the BCGA population, with a mutation density of only 18.4. In the BCGA cohort, D-A (57) and D-B (39.2) showed an increase in the mutation density compared to TCGA. The Chi-square test confirmed this difference as extremely significant (*p* < 0.0001) (Figure 3E). These results on the distribution of mutations across the domains suggest that the mutations that may promote tumorigenic conditions are specific to certain domains, which may lead to increased cancer severity. The study findings also indicate that the mutation burden significantly differs between TCGA and BCGA cohorts, highlighting cohort-specific variation in mutation density. These novel findings can reveal the reason behind the differences in the demographic profiles of breast cancer patients.

### 3.4. There Is a Significant Difference in the Position of the Pathogenic Mutations Between the TCGA and BCGA Breast Cancer Patient Populations

In the preceding section, we identified the hotspots or susceptible sites of mutations. It is important to note that there are various types of mutations, and not all mutations result in cancer development. Only mutations classified as likely pathogenic or pathogenic contribute to cancer severity. So, we analyzed the distribution of pathogenic mutation density within the FDs of *BRCA1* and its interacting genes in breast cancer using the TCGA and BCGA datasets. The analysis highlighted distinct patterns of pathogenic mutation density across key genes involved in breast cancer in these cohorts (Figure 4). The overall pathogenic mutations in the FDs of the *BRCA1* gene were lower in the TCGA cohort (Supplementary data Sheet 3). The RD in the BCGA cohort showed a pathogenic mutation density of 20.3 compared to zero in TCGA, although this specific difference did not reach statistical significance. Furthermore, the BCGA cohort displayed a distinct accumulation of pathogenic mutations in the BRCT1D (16.6) and BRCT2D (12.5) compared to TCGA, whereas TCGA showed significant pathogenic mutation density in the BRCT-AD (4.6) compared to only 0.2 mutation density in the BCGA samples (Figure 4A). For PALB2, both populations showed relatively lower pathogenic mutation densities in the FDs studied. The TCGA cohort exhibited a mutation density of 10.6 in the WD40D domain, compared to 5.9 in the BCGA cohort. While BCGA showed minor activity in the ChAMD and MRG15BD domains, these variations were not statistically significant (Figure 4B). A notable contrast was observed in the *RAD50* gene. Pathogenic mutations were almost exclusively localized to the BCGA cohort within the Zinc domain and MRE11-IS, showing a mutation density of 12.5 and 12.8, respectively. In contrast, the TCGA cohort showed no pathogenic mutation burden across any of the analyzed domains for this gene (Figure 4C). The *BARD1* gene revealed a BCGA cohort-specific prevalence of mutations extracted from the Indian population. While the TCGA dataset recorded zero pathogenic mutations in these domains, the BCGA cohort showed significant clustering in the RD, with a mutation density of 14.1 (Figure 4D). In the *RB1* gene, the mutation profile was mostly polarized to D-A in both of the cohorts. Furthermore, while both groups showed mutations in D-A, the BCGA mutation density of 17.2 was higher than that of the TCGA cohort of 12.20. The BCGA cohort also showed a reasonable mutation burden in D-B with a mutation density of 9.8, which was completely absent in the TCGA cohort (Figure 4E).

These findings, supported by Fisher’s exact test, show that pathogenic mutation hotspots vary significantly between cohorts; domains that lacked pathogenic enrichment in TCGA showed appreciable mutation density in BCGA after normalization (Supplementary data Sheet 4). For PALB2-WD40, BCGA had more non-pathogenic mutations compared to TCGA [odds ratio = 2.250 (95% CI: 0.09610 to 46.83), relative risk = 1.625]. The same was observed for RB1:D-A [odds ratio = 3.469 (95% CI: 0.6422 to 20.50), relative risk = 1.988] and BRCA1-UNK [odds ratio = 2.600 (95% CI: 0.1154 to 52.59), relative risk = 1.800]. On the contrary, BCGA had relatively more pathogenic mutations in RB1: UNK compared to TCGA [reciprocal odds ratio = 0.5000 (95% CI: 0.09449 to 2.397); reciprocal of relative risk = 0.5833]. In the case of both BRCA1-BRCT1 and BRCT2 domains, BCGA had a higher number of pathogenic mutations compared to its TCGA counterparts (reciprocal odds ratio = 0.2564 (95% CI: 0.01912 to 1.877) and 0.5000 (95% CI: 0.02595 to 9.793), respectively, and reciprocal of relative risk = 0.4423 and 0.7500, respectively). However, the most striking difference was observed for BRCA1-BRCT-AD, where TCGA had significantly more pathogenic mutations than BCGA [Fisher’s exact test, adjusted *p*-value = 0.0076; odds ratio = 9.58 (95% CI: 1.802 to 39.68), relative risk = 6.3]. We note here that, in the case of certain domains such as RD and BRCT1D, BRCT2D in BRCA1, C-CD and ZincD in RAD50, and RD and BRCT1D in BARD1, BCGA had a high number of pathogenic mutations in the BCGA cohort, but due to the absence of pathogenic mutations in these domains in the TCGA cohort, no statistical assessment was possible.

The significant differences in the domain mutation densities between the cohorts underscore the importance of considering population-specific mutational landscapes to better understand the molecular mechanisms driving breast cancer and justify that localized genetic screening is crucial for effective diagnosis and treatment.

**Figure 4:**
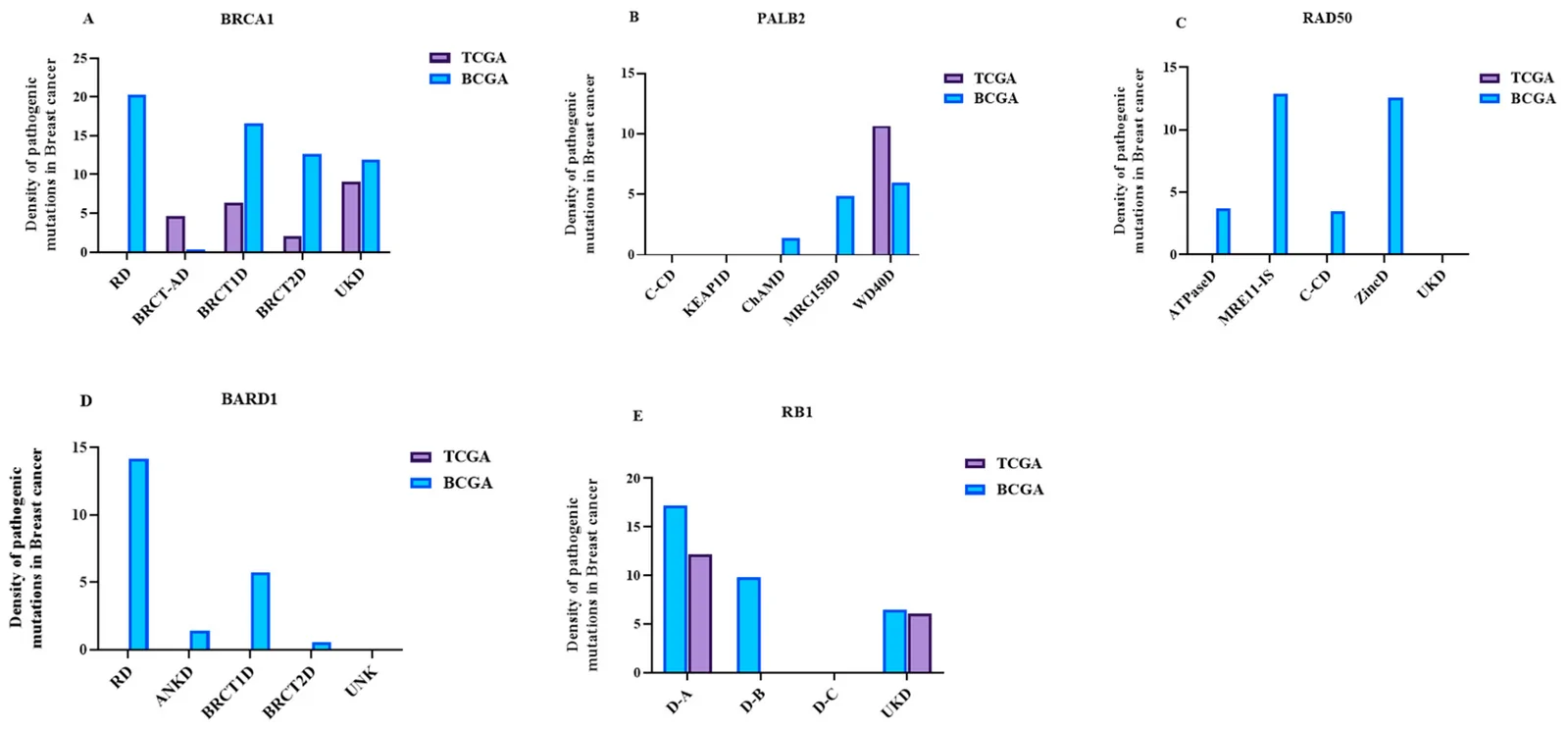
Domain-wise distribution of pathogenic mutations in breast cancer in TCGA and BCGA datasets. (**A**) *BRCA1*: Density of pathogenic mutations in RD, BRCT-AD, BRCT1D, BRCT2D, and UKD (TCGA *n* = 8, BCGA *n* = 119). (**B**) *PALB2*: Density of pathogenic of mutations in C-CD, KEAP1D, CHAM, MRG15BD, and WD40D (TCGA *n* = 1, BCGA *n* = 7). (**C**) *RAD50*: Density of pathogenic mutations in ATPaseD, MRE11BS, C-CD, ZincD, and UKD (TCGA *n* = 0, BCGA *n* = 9). (**D**) *BARD1*: Density of pathogenic mutations in RD, ANKD, BRCT1D, BRCT2D, and UNK (TCGA *n* = 0, BCGA *n* = 6). (**E**) *RB1:* Density of pathogenic mutations in D-A, D-B, D-C, and UKD (TCGA *n* = 5, BCGA *n* = 28). Bars show the proportion of pathogenic variants per kb for TCGA (purple) and BCGA (blue).

### 3.5. Cohort-Specific Distribution of Clinically Annotated Pathogenic Variants at Recurrent Mutation Sites Across TCGA and BCGA

In the previous section, we observed a cohort-specific shift in intrinsic domain susceptibility towards pathogenic mutation. Further analysis reveals a very interesting variation in the type of mutations between the cohorts, followed by pathogenicity (Figure 5). The most interesting data came from the cell cycle gene *RB1*, the *BRCA1* interactor, and the *BRCA1* gene itself. Notably, the frequency and distribution of clinically annotated pathogenic variants at recurrent mutation sites differed between the TCGA and BCGA cohorts.

In the case of *BRCA1*, a G-to-V mutation at position 1788 aa is pathogenic in the TCGA cohort, whereas changing from G to D, a different variant, at the same position in the BCGA data does not show any pathogenicity. Also, there are some positions in both cohorts that may undergo mutations, such as the substitution of glutamine at position 1811 with leucine, which are non-pathogenic in both cohorts. Frameshift mutations leading to different variants, such as E23Vfs17 and E23Sfs8, occur frequently in both TCGA and BCGA datasets but do not confer pathogenicity consistently. Some positions may be marked as highly vulnerable in the progression of breast cancer in the BCGA cohort from the Indian population. Notably, several substitutions in the BRCT1 domain—R1699L, C1697F, A1708E, and A1708V—are consistently pathogenic and frequently reported. A similar trend is observed in the BRCT2 domain, where mutations at positions S1715, M1775, and A1752, and missense mutations like D1739Y, G1770V, and R1835L are both recurrent and predominantly pathogenic. Some contrasting effects were also observed at various positions within the BCGA cohort. Mutation type also influences pathogenicity at specific residues. For example, at position 44 within the RING domain, a nonsense mutation (C44*) is non-pathogenic, while other alterations at the same residue are associated with pathogenicity. Similarly, a nonsense mutation at position G1738 (G1738*) is non-pathogenic, whereas a missense substitution at the same site (G1738V) is pathogenic. Likewise, the W1837C variant is frequently observed but exhibits variable pathogenicity across the dataset, which warrants cohort-specific mutation studies. Recurrent missense mutations, including P968Q, M1628, and M1652I, are observed within the BRCT1-associated and BRCT1 domains but show no pathogenicity. Collectively, these findings highlight that both the nature of the amino acid substitution and the cohort-specific context influence mutation pathogenicity. Importantly, the region spanning residues 1697 to 1775 within the BRCT domains in BCGA appears particularly sensitive to variation, suggesting that alterations in this segment are highly deleterious and may play a critical role in breast cancer progression (Figure 5A).

In the case of *RB1*, there are several common position mutations, but the pathogenicity varies with different variants between the two cohorts from different populations. At positions like R455, a nonsense mutation is pathogenic in the TCGA population at a significant frequency, whereas the same nonsense mutation at similar positions in the BCGA cohort is non-pathogenic. Notably, when arginine (R) is substituted by leucine (L) at the same position in the BCGA cohort, the mutation becomes pathogenic. So, this position (R455*/R455L) stands out as recurrent and pathogenic in both cohorts, with both nonsense and missense mutations. This suggests that residue R455 represents a critical functional site within D-A, where both truncating and amino acid-altering mutations can significantly impact protein function. There are a few other positions, like R552, where a nonsense mutation brings in pathogenicity in the TCGA population. Interestingly, when arginine (R) is substituted by leucine (L), the missense mutations in the BCGA cohort no longer remain pathogenic (Figure 5B). There are also a few reverse incidences in the BCGA population. Nonsense mutations at position R445 are non-pathogenic in the BCGA population. The same positions that undergo a substitution mutation with leucine (L) in the same cohort become pathogenic. These observations further highlight that in-depth studies on cohort/population-specific mutations are required to validate these findings, as ClinVar classification is not a cohort-specific property. A few positions in BCGA remain non-pathogenic, though they are present at high frequency (R661W, freq. = 3.8, R621S, freq. = 2.9, R556, freq. = 5.8) or substituted by different aa (R579L or R579G), (Figure 5B).

Distinct mutation patterns were observed in the *BARD1* gene, with no pathogenic or non-pathogenic mutations in the TCGA cohort, while the BCGA cohort showed pathogenic mutations in the Ring and BRCT2 domains, the same as *BRCA1*, emphasizing the functional importance of Ring and BRCT domains across the genes (Figure 5C). In the case of the *RAD50* gene, though non-pathogenic mutations are present in the TCGA data, there were no pathogenic mutations in the TCGA BC data. In the BCGA population, the pathogenic mutations were mostly housed in the CC-Zinc domain (Figure 5D).

## 4. Discussion

The present study investigates the mutational landscape of the *BRCA1* gene and its interacting partners in different cohorts of breast cancer patients extracted from demographically different populations. The analysis using TCGA patient data from the COSMIC database confirms the differences in the prevalence of mutations in the *BRCA1* gene and its interactor genes, *PALB2*, *RAD50*, *RB1*, and *BARD1*, in cancer samples compared to normal tissue samples, signifying their potential role in cancer pathogenesis. The percentage of mutations in housekeeping genes in cancer and normal samples was significantly low (Figure 1). We also observed that it is not the number of gene mutations but the site of mutation that plays an important role in tumorigenesis. The current findings were consistent with existing literature, which shows an increase in mutations in cancer conditions [2,3,17,18,39,40]. Further in-depth analysis with the TCGA and BCGA datasets identified that the mutations in the cancer samples are functional domain-specific; moreover, in all of the genes *BRCA1*, *PALB2*, *RAD50*, *RB1*, and *BARD1*, the distribution of mutations appears to be heterogeneous across FDs, leading to the identification of specific mutationally susceptible sites in the cancer-causing oncogenes, which change between cohorts that differ demographically. These specific mutation hotspot sites directly correlate with the development of pathogenicity in the disease condition. The severity of breast cancer and the frequency of mortality from breast cancer in the Indian population are higher compared to the Western population [41]. A similar conclusion can be drawn through this work from the analysis of the abundance of pathogenic mutations in most of the *BRCA1*-interacting genes. We identified the BRCT-1D in the case of *BRCA1*, the WD-40 D in *PALB2*, and D-A in *RB1* as the target sites that have more potential to develop into pathogenic conditions upon mutations in the TCGA cohort. On the other hand, in the BCGA cohort, in a portion of the Indian population, except for WD-40 D and D-A in the *PALB2* and *RB1* genes, respectively, the pathogenic profile changes completely in the *BRCA1*, *BARD1*, and *RAD50* genes. Mutation sites like ZD in *RAD50* and RD in both BRCA1 and *BARD1* play a role in pathogenicity. Hence, the difference in severity in the breast cancer conditions between the two population-specific cohorts may be due to differences in the mutation profile, which contributes to pathogenicity [42,43] (Figure 4).

The functional analysis of the domains from previous literature justifies that the mutation-susceptible sites that lead to more pathogenic conditions serve the function of maintaining genetic integrity (Figure 6). Compromise in genetic integrity is the cause of the development of more severe conditions [44]. We also speculate that the mutations linked to the DNA damage repair response can lead to more severe conditions. In the case of *BRCA1* in the TCGA cohort, the BRCT-AD has more pathogenic mutations (Figure 4A), whereas in the Indian population, the pathogenic mutations are high in the RD. In the case of *PALB2*, the WD40 domain has the highest pathogenic mutations in both cohorts, as the independent function of the WD40-D helps in the interaction with DNA polymerase η, which is vital for the initiation of HR-mediated DNA synthesis [45] (Figure 4B). The ChAM-D and MRG15-BD are also mutated in the BCGA cohort, as the *PALB2* chromatin association is dependent on both ChAM-D and MRG15–BD [46]. The mutation at MRG15-BD is lethal, and the mutations in both MRG15-BD and ChAM-D show a synergistic effect (Figure 6).

In the case of the *RB1* gene, the distribution of pathogenicity between the two cohorts is changing significantly. The TCGA and BCGA cohorts have the highest density of pathogenic mutations in D-A, but D-B also plays a considerable role in pathogenesis in the BCGA population. In *RB1*, all three domains, domains A, B, and C, play a role in cell cycle checkpoint activities. Phosphorylated D-A and D-B, in the presence of D-C, dimerize for E2F activation [40,45]. In the TCGA population, it is mostly the nonsense mutations in D-A that are pathogenic. In contrast, a nonsense mutation in the same position in the BCGA population does not turn out to be pathogenic. Furthermore, the BCGA population shows higher pathogenicity with D-B mutations, and D-C plays a lesser role in developing pathogenicity. These observations suggest possible functional divergence that warrants experimental validation. In the TCGA cohort, as missense mutations in D-A only led to pathogenicity, D-A is important for the functioning of *RB1*. But in the BCGA population, many missense mutations in the same position of D-A do not lead to a pathogenic condition, whereas mutations in D-B add pathogenicity. This shows that both D-A and D-B are important in regulating the role of the RB1 protein.

In the case of other *BRCA1* interactors, like *BARD1* and *RAD50*, pathogenic mutations were observed only in the BCGA population. The mutations in the RD and the ZD, respectively, are highly likely to confer pathogenicity. This again explains the difference in the severity of the cancer condition in the BCGA population compared to the TCGA populations.

This study has several limitations that should be considered when interpreting the findings. First, the TCGA and BCGA cohorts differ in sample size, sequencing platforms, mutation-calling pipelines, sample selection criteria, and availability of clinical metadata. The relatively smaller BCGA cohort may also result in greater sampling variability and reduced statistical power, particularly for infrequently mutated genes or small functional domains. These factors may contribute to the observed differences in mutation frequencies and domain-level distributions. Therefore, the findings should be interpreted as exploratory cohort-level comparisons rather than definitive evidence of population-specific biological differences.

Second, the present analysis integrates somatic mutation data with independent clinical variant annotations. ClinVar classifications were treated as variant-level annotations and were not considered cohort-specific. Variants lacking a pathogenic or likely pathogenic annotation were not assumed to be benign, and clinical annotation alone does not establish the functional effect of a variant in the tumors analyzed. Functional studies will therefore be required to determine the biological significance of the observed domain-level patterns.

Third, domain-length normalized mutation density was primarily used for descriptive comparison and visualization of mutation distributions. Although statistical tests were applied to compare observed mutation frequencies, the relatively small number of mutations in some genes or domains, together with differences in cohort size and composition, limits the strength of conclusions from individual domain-level comparisons. Future studies using larger and more balanced cohorts should provide more precise estimates of these associations.

Finally, this study does not include clinical outcome measures such as overall survival, progression-free survival, tumor stage (Supplementary data Sheets 5 and 6), molecular subtype, recurrence, or treatment response. Consequently, the observed differences in mutation distribution cannot be used to establish differences in disease severity, prognosis, recurrence risk, or therapeutic response between TCGA and BCGA. Similarly, mutation-enriched domains or recurrent sites should not be considered confirmed cancer-driver regions or therapeutic targets without functional validation. Larger, harmonized cohorts incorporating clinical and functional data will be important for determining the biological and clinical relevance of these observations.

## 5. Conclusions

The current study provides an exploratory analysis of the domain-level distribution of somatic mutations in *BRCA1* and its interacting genes *PALB2*, *RAD50*, *BARD1*, and *RB1* across the TCGA and BCGA breast cancer cohorts. The observed mutations were not uniformly distributed across the proteins, with differences in the distribution of variants across functional and non-characterized regions between the two cohorts. In addition, variant-level clinical annotations identified pathogenic and likely pathogenic variants occurring within several functional domains.

These findings suggest that consideration of protein-domain context may provide additional information beyond gene-level mutation status when characterizing the mutational landscape of breast cancer. However, the observed differences between TCGA and BCGA should be interpreted as cohort-level differences in the distribution of somatic variants rather than as evidence of population-specific biological mechanisms or differences in disease severity. The two cohorts differ in sample size, clinical composition, sequencing approaches, and data-generation and variant-calling procedures, which may contribute to the observed patterns.

Importantly, this study does not include clinical outcome measures such as tumor stage, molecular subtype, survival, recurrence, or treatment response and therefore cannot establish associations between the observed domain-level mutation patterns and disease severity, prognosis, or therapeutic efficacy. Similarly, the identification of clinically annotated pathogenic or likely pathogenic variants does not by itself establish their functional effects within the tumors analyzed. The present findings should therefore be considered hypothesis-generating. Larger, harmonized cohorts together with functional validation and clinical outcome data will be required to determine whether the observed domain-level patterns have biological or clinical relevance.

## Figures and Tables

**Figure 1:**
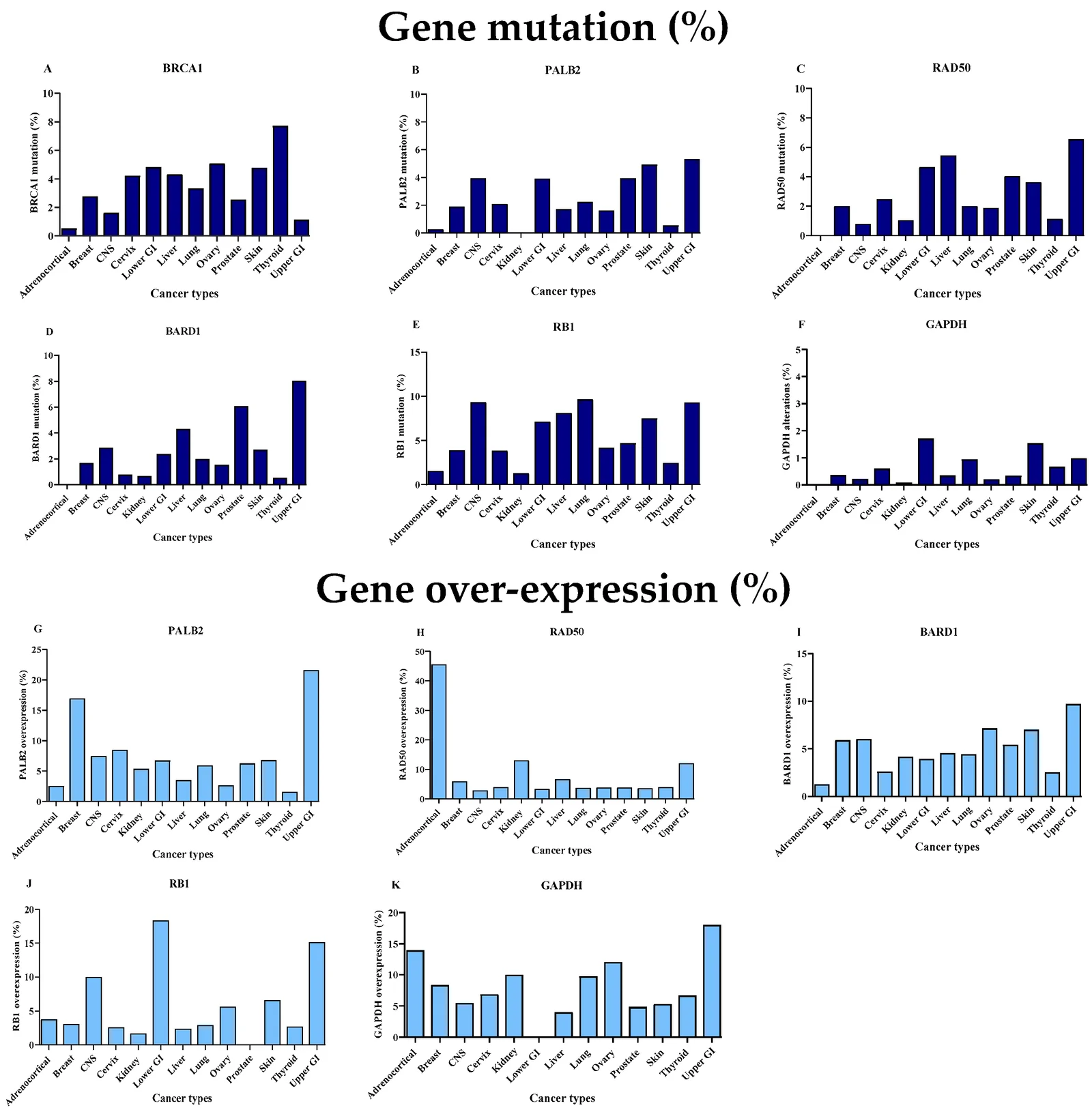
Elevated mutation burden and altered oncogene expression are observed across various cancer types. (**A**–**F**) Dark blue bars indicate the percentage of mutations in the oncogene *BRCA1*, and its interactors *PALB2*, *RAD50*, *BARD1*, and *RB1*, along with the housekeeping *GAPDH* gene in CSs. (**G**–**K**) Light blue bars represent the percentage of gene expression in *BRCA1* interactors *PALB2*, *RAD50*, *BARD1*, and *RB1*, and the housekeeping *GAPDH* within CSs. The *X*-axis categorizes various cancer types, and the *Y*-axis quantifies the percentage (%) of gene alterations. Data were obtained from the COSMIC database.

**Figure 2:**
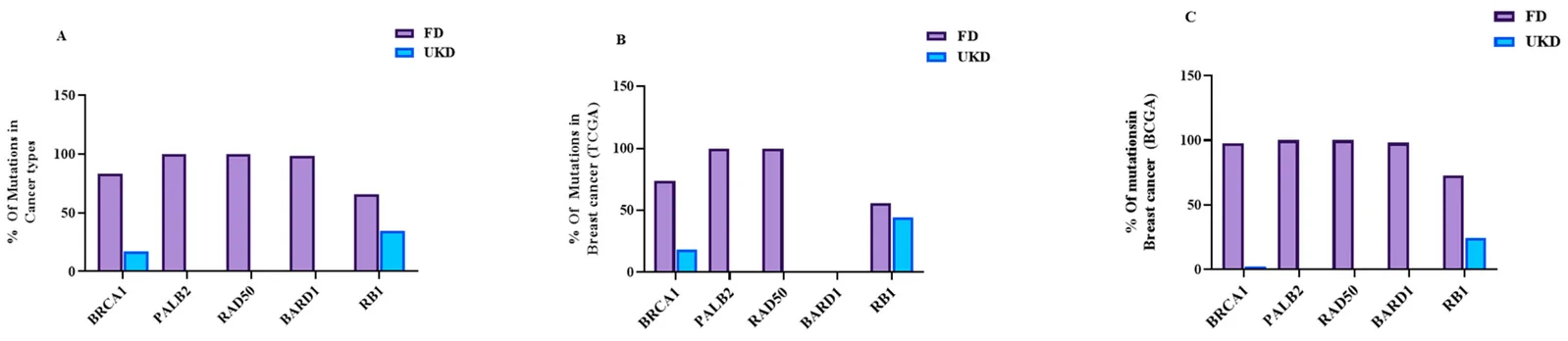
Distribution of mutations across cancer types and breast cancer cohorts. (**A**) Proportion of mutations in the defined FD versus UKD for *BRCA1* (*n* = 292), *PALB2* (*n* = 169), *RAD50* (*n* = 236), *BARD1* (*n* = 141), and *RB1* (*n* = 444) across multiple cancer types (bladder, brain, breast, cervical, colorectal, B-cell lymphoma, esophageal, glioblastoma, head and neck, kidney, liver, lung, ovarian, pancreatic, prostate, sarcoma, stomach, skin, thyroid and uterine cancers), screened from the TCGA dataset (*n*= 10,967). (**B**) Proportion of breast cancer mutations in BRCA1 (*n* = 25), *PALB2* (*n* = 7), *RAD50* (*n* = 10), *BARD1* (*n* = 0), and *RB1* (*n* = 27) in TCGA mapped to FD and UKD for each gene and (**C**) corresponding distribution of breast cancer mutations in BRCA1 and its interactors in the BCGA dataset in BRCA1 (*n* = 249), *PALB2* (*n* = 50), *RAD50* (*n* = 33), *BARD1* (*n* = 102), and *RB1* (*n* = 52).

**Figure 3:**
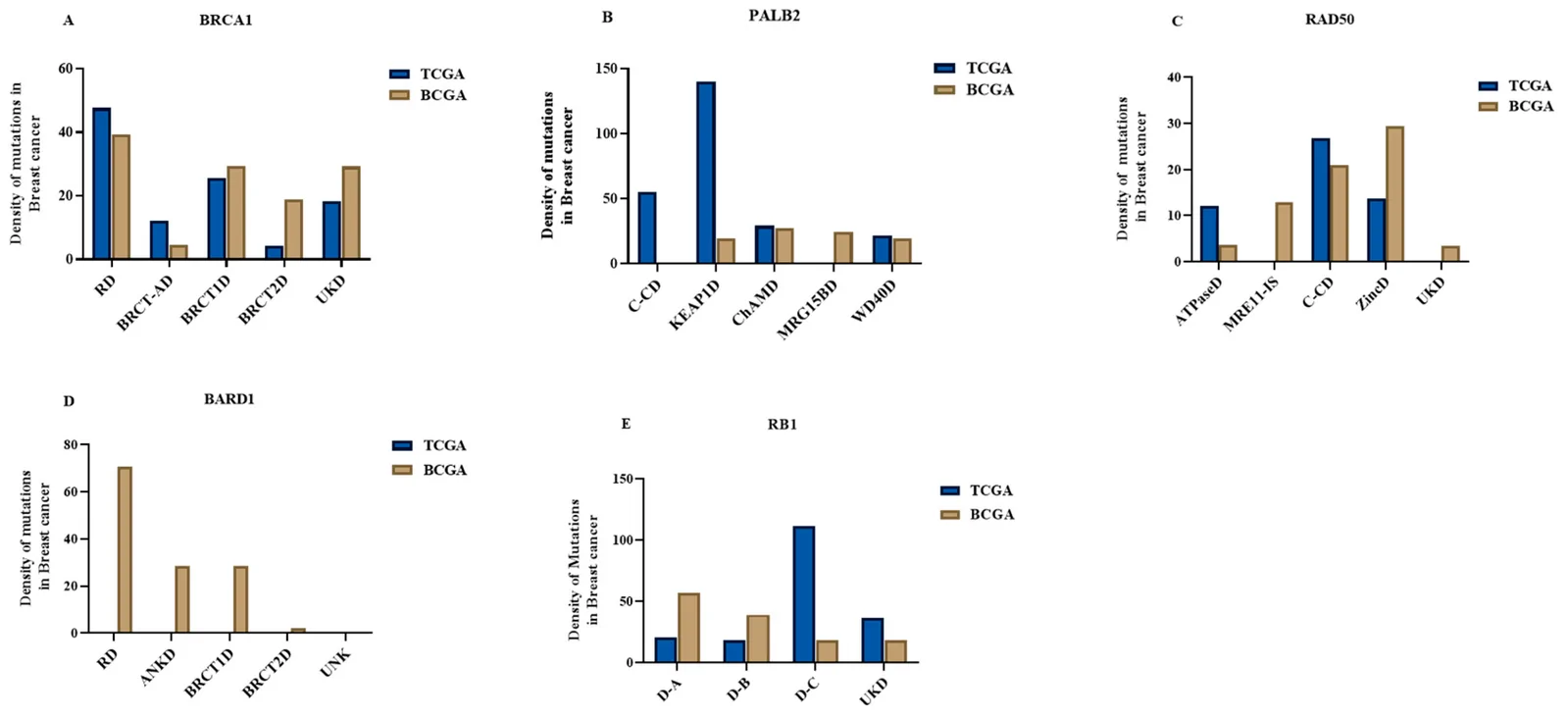
Domain-wise distribution of mutations in breast cancer samples from the TCGA and BCGA datasets differs significantly. (**A**) Domain-wise distribution of mutations in breast cancer samples from the TCGA and BCGA cohorts. (**A**) BRCA1: density of mutations in breast cancer (TCGA *n* = 25, BCGA *n* = 249) mapped to the RD, BRCT-AD, BRCT1D, BRCT2D, and UKD domains. (**B**) PALB2: density of mutations (TCGA *n* = 7, BCGA *n* = 50) in the C-CD, KEAP1D, MRG15BD and WD40D domains. (**C**) RAD50: mutation rate (TCGA *n* = 10, BCGA *n* = 33) across the ATPaseD, MRE11BS, C-CD, ZincD, and UKD domains. (**D**) BARD1: density of mutations (TCGA *n* = 0, BCGA *n* = 52) within the RD, ANKD, BRCT1D, BRCT2D, and UKD domains. (**E**) RB1: density of mutations (TCGA *n* = 27, BCGA *n* = 102) across the D-A, D-B, D-C, and UKD domains. Bars represent the density of mutations in each functional domain for the TCGA (blue) and BCGA (brown) cohorts.

**Figure 5:**
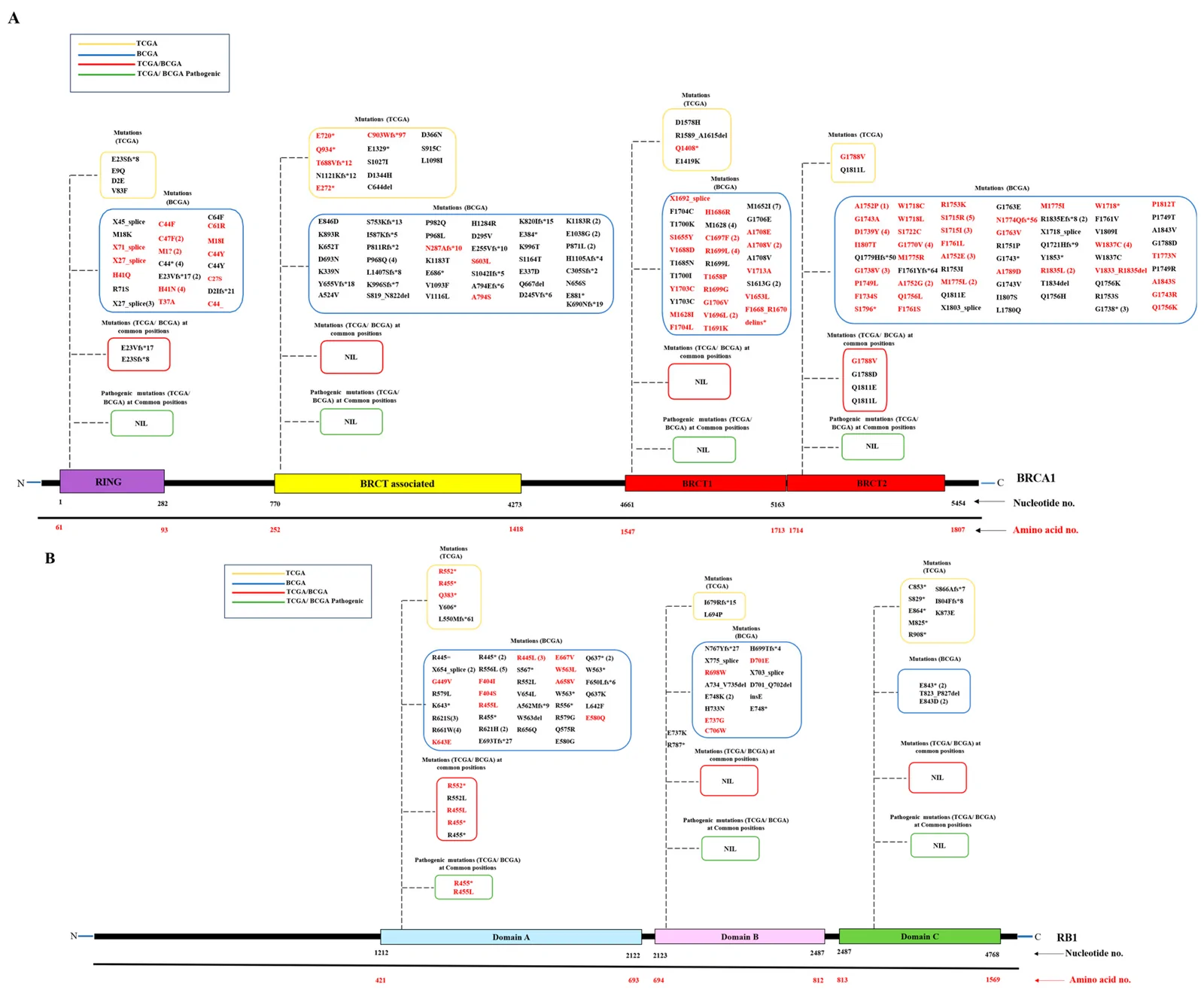

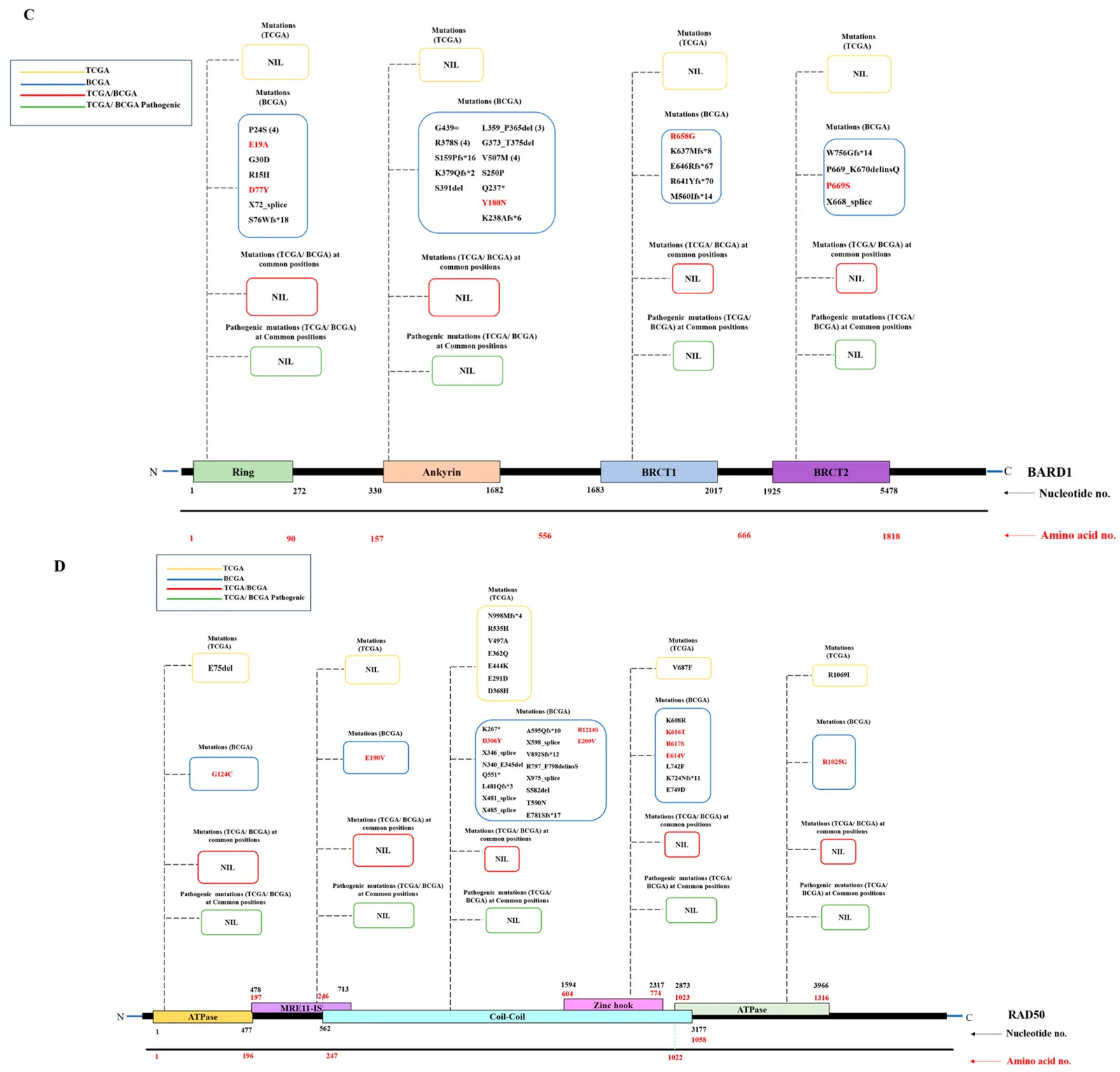
Significant functional differences of the mutations in the same positions between and within the cohorts. Schematic representation of the distribution of variants identified in the TCGA and BCGA breast cancer cohorts along the full-length BRCA1 protein and its interactors. The protein illustration shows the relative positions of the domains, with nucleotide and amino acid coordinates indicated below. Boxes above the protein track summarize, for each region, all mutations observed in TCGA (yellow), BCGA (blue), shared TCGA/BCGA variants (red), and shared pathogenic variants (green), with “NIL” denoting the absence of variants in that category at the indicated region; within the mutation boxes, variants highlighted in red font correspond to those classified as pathogenic based on clinical and in silico evidence. (**A**) Distribution of *BRCA1* variants on the protein diagram showing relative positions of the N-terminal RD, central BRCT-AD, and C-terminal BRCT1D and BRCT2D. (**B**) Distribution of *RB1* variants along the full-length RB1 protein. The linear protein diagram shows the relative positions of the N-terminal D-A, central D-B, and D-C. (**C**) Distribution of *BARD1* variants along the full-length BARD1 protein. The linear protein diagram shows the relative positions of the RD, ANKD, BRCT1D, and BRCT2D. (**D**) Distribution of *RAD50* variants along the full-length RAD50 protein. The linear protein diagram shows the relative positions of the N-terminal ATPaseD, MRE11-IS, extended C-CD, ZincD, and C-terminal ATPaseD. * Signifies nonsense mutation (a mutation that makes it a stop codon).

**Figure 6:**
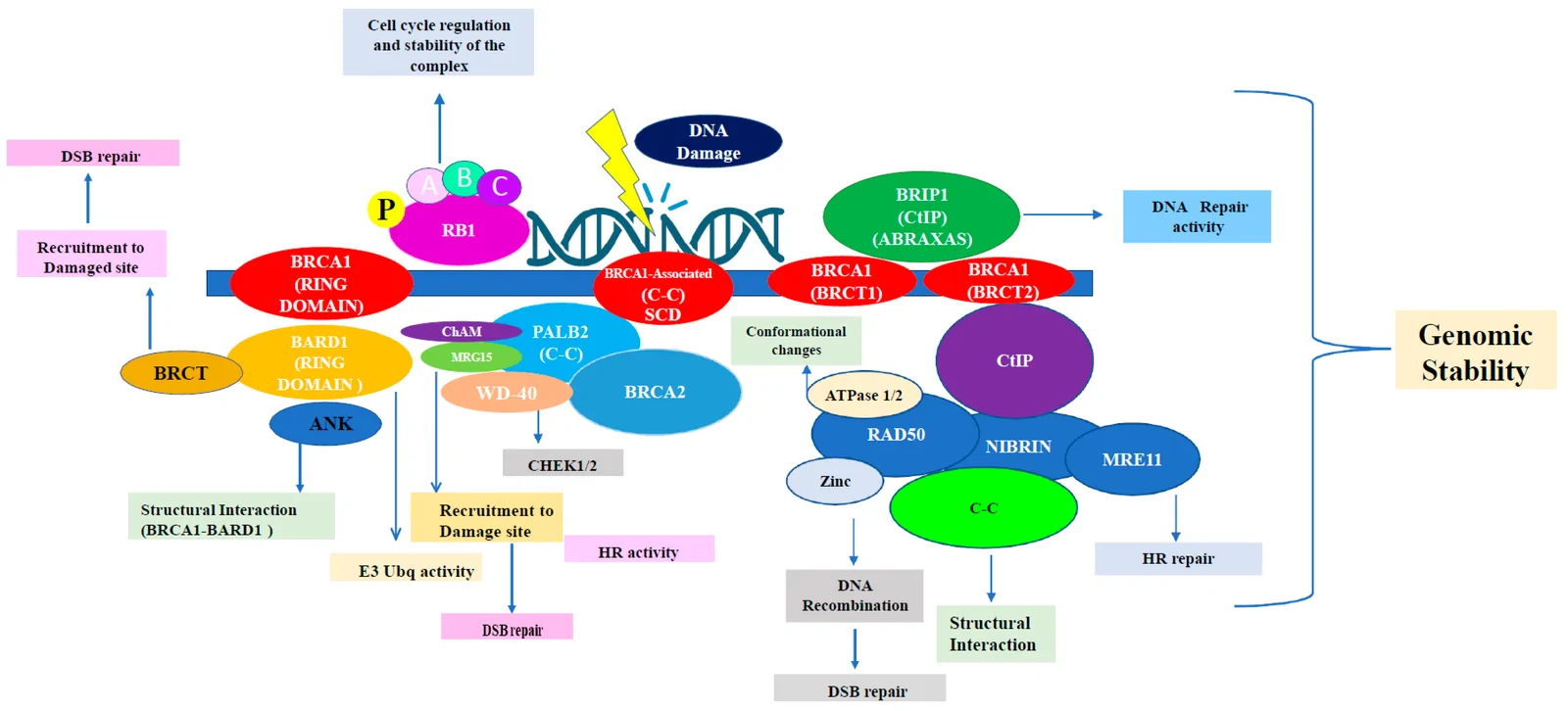
Schematic representation and roles of BRCA1 and associated proteins in homologous recombination-mediated DNA double-strand break repair and maintenance of genomic stability. Following DNA damage, BRCA1 is recruited to breaks, where its N-terminal RING domain forms an E3 ubiquitin ligase complex with BARD1, its central coiled-coil (C-C) motif interacts with PALB2 to bridge BRCA1 and BRCA2, and its C-terminal BRCT repeats bind phosphorylated partners such as BRIP1, CtIP, and ABRAXAS (A, B, and C complexes) to coordinate DNA end resection, checkpoint activation, and repair pathway choice. PALB2 (through ChAM, MRG15, and WD40 regions) connects BRCA2 and RAD51 to damaged chromatin, whereas the MRN complex components RAD50 (ATPase, coiled-coil, and zinc hook domains), MRE11, and Nibrin (NBS1) promote DNA end processing, recombination, and structural stabilization of repair foci, collectively ensuring efficient homologous recombination, double-strand break repair, and preservation of genomic stability.

**Table 1:** Exon and domain mapping of *BRCA1* and its key interactors.

Gene	Functional Domains (FDs)	Nomenclature	Exon	Amino Acid Position	Domain Size (KB)	Unknown Domains (UKDs)	Domain Size (KB)	Amino Acid Position
*BRCA1*	Ring domain	RD	Exon 1–2	61–93	0.336	Exon 3–7	0.442	94–237
	BRCA1 C-Terminal Associated Domain (serine cluster domain, coil-coil domain)	BRCT-AD (SCD, C-CD)	Exon 8–10	252–1418	4.318			
	BRCT1 domain	BRCT1D	Exon 13–15	1547–1713	0.628			
	BRCT2 domain	BRCT2D	Exon 16–22	1714–2347	1.914			
*PALB2*	Coil-coil domain	C-CD	Exon 1–2	66–85	0.260	NA	NA	NA
	Kelch-like ECH-associated protein 1	KEAPLD	Exon 3	86–119	0.102			
	Chromatin-association motif	ChAMD	Exon 4	120–609	1.472			
	MRG15	MRG15BD	Exon 5	610–885	0.829			
	Tryptophan-Aspartic Acid 40 Repeats	WD40D	Exon 6–13	886–1326	1.34			
*RAD50*	Adenosine Triphosphatase Domain	ATPaseD	Exon 1–2, Exon 16–23	1–196 1023–1316	0.56 1.093	Exon 24–25	4.3	1360–2750
	Meiotic Recombination11 Interaction Site	MRE11-IS	Exon 2–3	197–246	0.235			
	Coil-coil domain	C-CD	Exon 3–17	247–1022	2.61			
	Zinc hook	ZincD	Exon 9–12	604–774	0.723			
*BARD1*	Ring domain	RD	Exon 1	90	0.272	Exon 2	0.05	108
	Ankyrin domain	ANKD	Exon 3–6	157–556	1.353			
	BRCT1 domain	BRCT1D	Exon 7–9	557–666	0.336			
	BRCT2 domain	BRCT2D	Exon 10–11	667–1818	3.554			
*RB1*	Domain A	D-A	Exon 11–19	421–693	0.911	Exon 1–10	1.211	99–396
	Domain B	D-B	Exon 20–22	694–812	0.4			
	Domain C	D-C	Exon 23–27	813–1569	0.266			

## Data Availability

All data analysed in this study were obtained from publicly available databases. TCGA breast cancer mutation data were retrieved through cBioPortal (https://www.cbioportal.org/) from the Breast Invasive Carcinoma (TCGA, PanCancer Atlas) study (study ID: brca_tcga_pan_can_atlas_2018; accessed on 14 September 2026). BCGA cohort data were retrieved through the BCGA cBioPortal (https://bcga.iitm.ac.in/) instance (accessed on 12 December 2025); the exact BCGA study identifier(s), query settings, cohort-selection criteria, and sample/patient information are provided in Appendix A Table A1. Exon mapping was based on NCBI RefSeq transcripts for BRCA1 (NM_001407594.1), PALB2 (NM_024675.4), RAD50 (NM_005732.4), BARD1 (NM_000465.4), and RB1 (NM_000321.3). COSMIC was used for mutation cross-referencing, where applicable. The reference genome build, protein-domain coordinates, transcript/exon annotations, query metadata, variant-filtering criteria, raw exported variant tables, processed analysis datasets, sample-size information, and complete Excel-based analytical workflow are available in Supplementary data (an Excel workbook comprising five worksheets) that contain the data supporting Figure 2, Figure 3 and Figure 4 and the BCGA and TCGA stage-wise analyses of all cancer samples, breast cancer mutations, and breast cancer pathogenic mutations. No new primary sequencing data were generated or deposited in a separate repository.

## Supplementary Materials

The following supporting information can be downloaded at https://www.mdpi.com/article/10.3390/cancers18193083/s1.

## Abbreviations

ATM	Ataxia-telangiectasia mutated
BARD1	BRCA-Associated Ring Domain 1
BCGA	Bharat Cancer Genome Atlas
BRCA1	Breast Cancer Gene-1
BRCA2	Breast Cancer Gene-2
BRIP1	BRCA1 Interacting Protein C-terminal Helicase 1
CH1	Cadherin 1
COSMIC	Catalogue Of Somatic Mutations in Cancer
CHEK2	Checkpoint kinase 2
DSBs	Double-Strand Break Repair
HBOC	Hereditary Breast and Ovarian Cancer
PALB2	Partner Localizer of BRCA2
PI3K	Phosphotidylinositol-3 Kinase
PTEN	Phosphatase and Tensin Homolog on Chromosome
RB1	Retinoblastoma 1
STK11	Serine/threonine Kinase 11
TCGA	The Cancer Genome Atlas
TP53	Tumor Protein p53

## Appendix A

**Table A1:** Exon and domain mapping of BRCA1 and its key interactors.

Gene	NCBI Transcript ID (Mapping Exon)	NCBI Reference Sequence ID	Isoform Type
*BRCA1*	Gene ID: 672	NM_001407594.1	Isoform-1
*PALB2*	Gene ID: 79728	NM_024675.4	Isoform-1
*RAD50*	Gene ID: 10111	NM_005732.4	Isoform-1
*BARD1*	Gene ID: 580	NM_000465.4	Isoform-1
*RB1*	Gene ID: 5925	NM_000321.3	Isoform-1
